# Supplementary material for: Holostean genomes reveal evolutionary novelty in the vertebrate immunoproteasome that have implications for MHCI function
Source: Mol Biol Evol. 2026 Feb 3;43(3):msag030. doi: 10.1093/molbev/msag030 (PMC12983459; doi:10.1093/molbev/msag030)
Supplement: msag030_Supplementary_Data [file msag030_supplementary_data.zip › Supplementary Figures 20250423.pdf]

— Supplementary Figures —

Holostean genomes reveal evolutionary novelty in the vertebrate immunoproteasome that have implications for MHCI function

Andi V. Barker[1], Kara B. Carlson[1,2], Dustin J. Wcisel[1], Ian Birchler De Allende[1,2,3], Ingo Braasch[4], Michael Fisk[5], Alex Dornburg[6†]*, Jeffrey A. Yoder[1,2,3,7†]*

1. Department of Molecular Biomedical Sciences, College of Veterinary Medicine, North Carolina State University, Raleigh, North Carolina, USA
2. Genetics and Genomics Academy, North Carolina State University, Raleigh, North Carolina, USA
3. Department of Biological Sciences, North Carolina State University, Raleigh, North Carolina, USA
4. Department of Integrative Biology, Michigan State University, East Lansing, Michigan, USA
5. Aquatic Wildlife Diversity Group, North Carolina Wildlife Resources Commission, Raleigh, North Carolina, USA
6. Department of Bioinformatics and Genomics, University of North Carolina at Charlotte, Charlotte, North Carolina, USA
7. Comparative Medicine Institute, North Carolina State University, Raleigh, North Carolina, USA

Table of Contents

Supplementary Figure S1. Bowfin PSMB8 sequences are F lineage. 2
Supplementary Figure S2. Gar PSMB8 sequences are A lineage. 4
Supplementary Figure S3. Alignment of select PSMB8 sequences. 7
Supplementary Figure S4. Thirty-first residues of PSMB8 types. 8
Supplementary Figure S5. Essential TAP1 residues from individual bowfin. 9
Supplementary Figure S6. Essential TAP1 residues from individual longnose gar. 10
Supplementary Figure S7. Essential TAP2 residues from individual bowfin. 11
Supplementary Figure S8. Essential TAP2 residues from individual longnose gar. 12
Supplementary Figure S9. Collection sites for bowfin and longnose gar. 13
References. 14

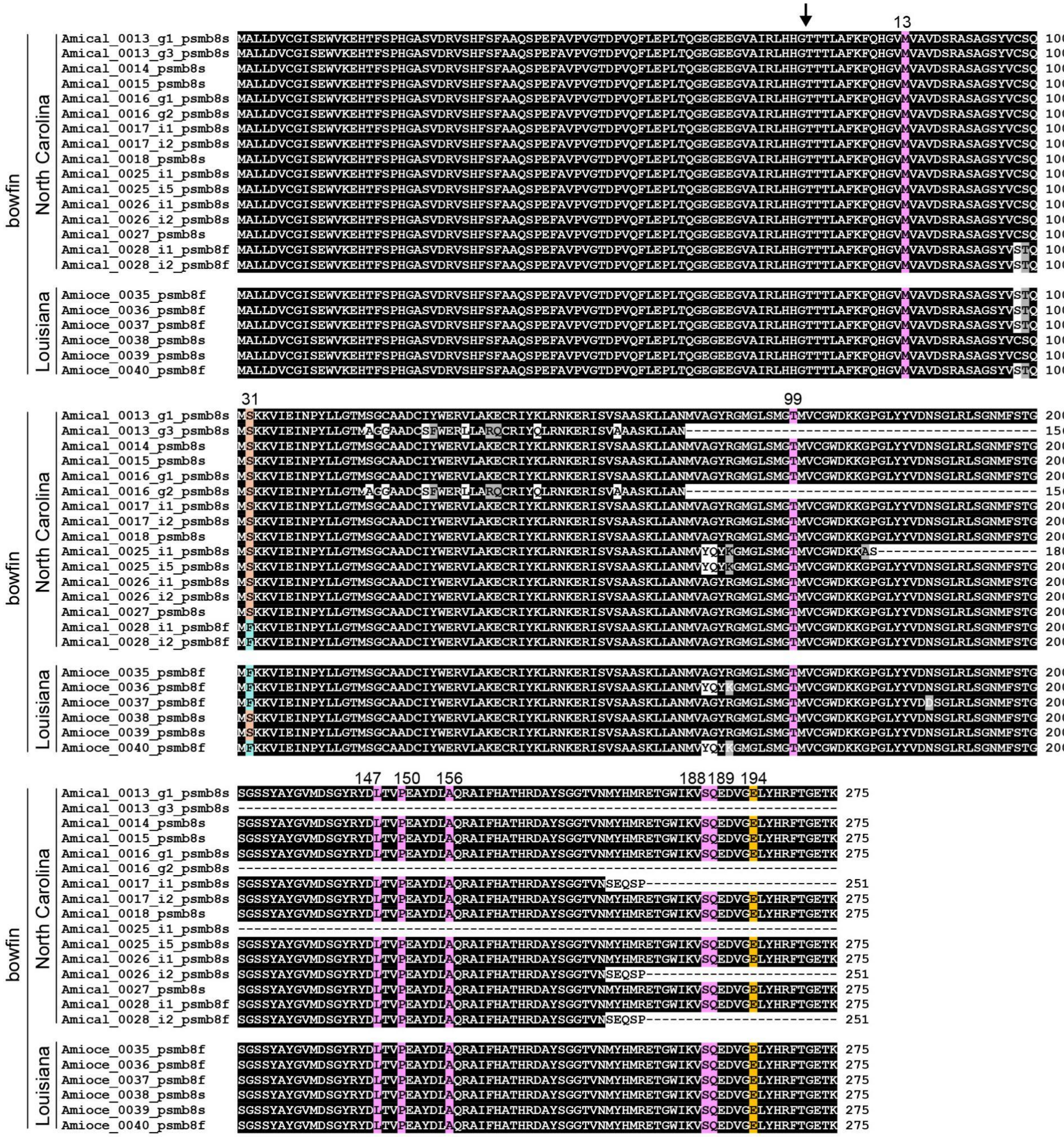

Supplementary Figure S1. Bowfin PSMB8 sequences are F lineage.

Full-length PSMB8 sequences from ruddy bowfin (Amical; North Carolina) and eyetail bowfin (Amioce; Louisiana) were aligned using Clustal Omega (Sievers and Higgins, 2021). Positions that are ≥70% identical are shaded black and those that are structurally related are shaded gray. The predicted cleavage site which produces the mature protein is indicated with an arrow. The eight residues described as predictive for the A and F lineages (Noro and Nonaka 2014) are shaded gold and pink, respectively. Residues that do not match either lineage are shaded blue. Residue 31, which defines the PSMB8 type is shaded orange (S type) or aqua (F type). All sequences and display identifiers are provided in **Supplementary Table S1.**

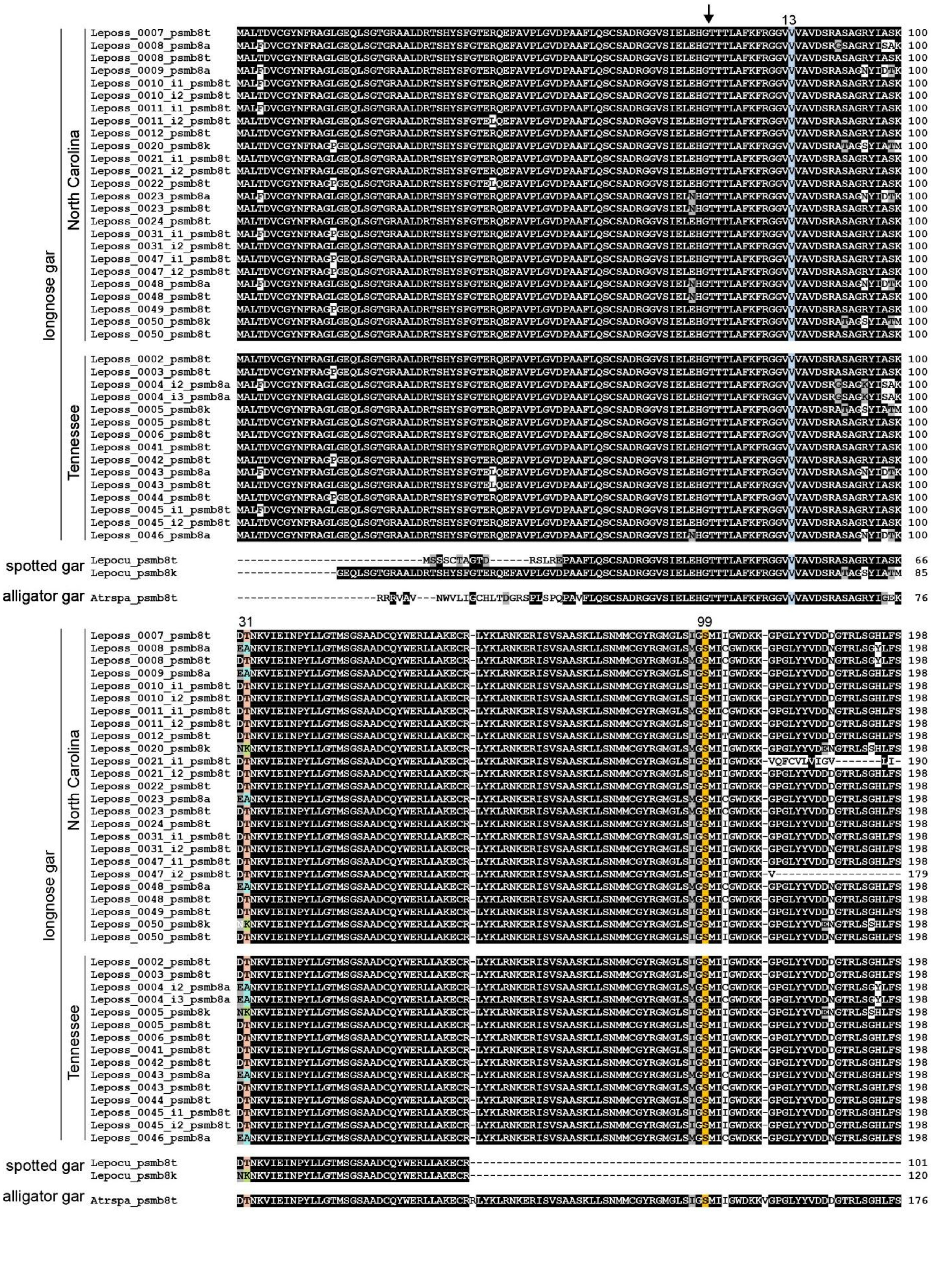

13
31
99
longnose gar
North Carolina
Tennessee
spotted gar
alligator gar
Leposs_0007_psmb8t
Leposs_0008_psmb8a
Leposs_0008_psmb8t
Leposs_0009_psmb8a
Leposs_0010_i1_psmb8t
Leposs_0010_i2_psmb8t
Leposs_0011_i1_psmb8t
Leposs_0011_i2_psmb8t
Leposs_0012_psmb8t
Leposs_0020_psmb8k
Leposs_0021_i1_psmb8t
Leposs_0021_i2_psmb8t
Leposs_0022_psmb8t
Leposs_0023_psmb8a
Leposs_0023_psmb8t
Leposs_0024_psmb8t
Leposs_0031_i1_psmb8t
Leposs_0031_i2_psmb8t
Leposs_0047_i1_psmb8t
Leposs_0047_i2_psmb8t
Leposs_0048_psmb8a
Leposs_0048_psmb8t
Leposs_0049_psmb8t
Leposs_0050_psmb8k
Leposs_0050_psmb8t
Leposs_0002_psmb8t
Leposs_0003_psmb8t
Leposs_0004_i2_psmb8a
Leposs_0004_i3_psmb8a
Leposs_0005_psmb8k
Leposs_0005_psmb8t
Leposs_0006_psmb8t
Leposs_0041_psmb8t
Leposs_0042_psmb8t
Leposs_0043_psmb8a
Leposs_0043_psmb8t
Leposs_0044_psmb8t
Leposs_0045_i1_psmb8t
Leposs_0045_i2_psmb8t
Leposs_0046_psmb8a
Lepocu_psmb8t
Lepocu_psmb8k
Atrspa_psmb8t

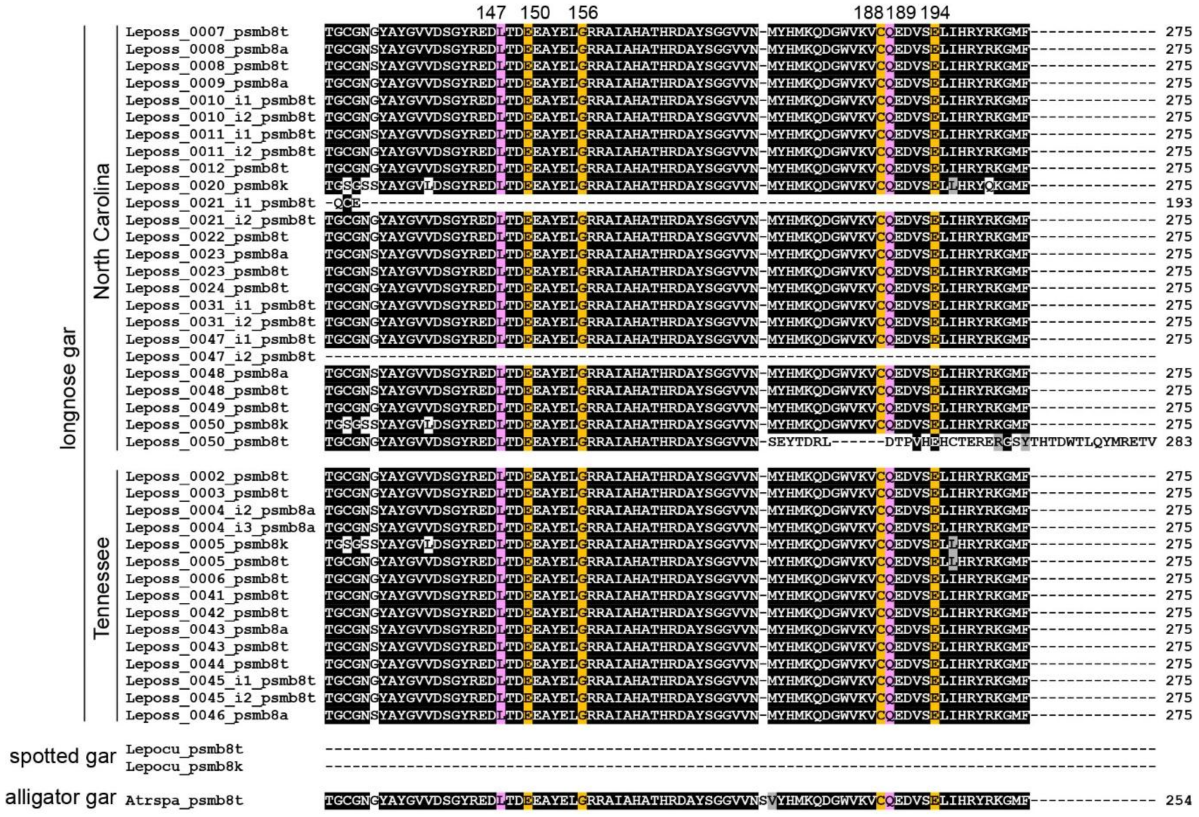

Supplementary Figure S2. Gar PSMB8 sequences are A lineage.

Full-length longnose gar (Leposs) PSMB8 sequences from North Carolina and Tennessee were aligned with reference sequences from spotted gar (Lepocu; Braasch et al. 2016) and alligator gar *(*Atrspa; Bi et al. 2021) using Clustal Omega (Sievers and Higgins 2021). Positions that are ≥70% identical are shaded black and those that are structurally related are shaded gray. The predicted cleavage site which produces the mature protein is indicated with an arrow. The eight residues described as predictive for the A and F lineages (Noro and Nonaka 2014) are shaded gold and pink, respectively. Residues that do not match either lineage are shaded blue. Residue 31, which defines the PSMB8 type is shaded orange (T type), aqua (A type) or green (K type). All sequences and display identifiers are provided in **Supplementary Table S2**.

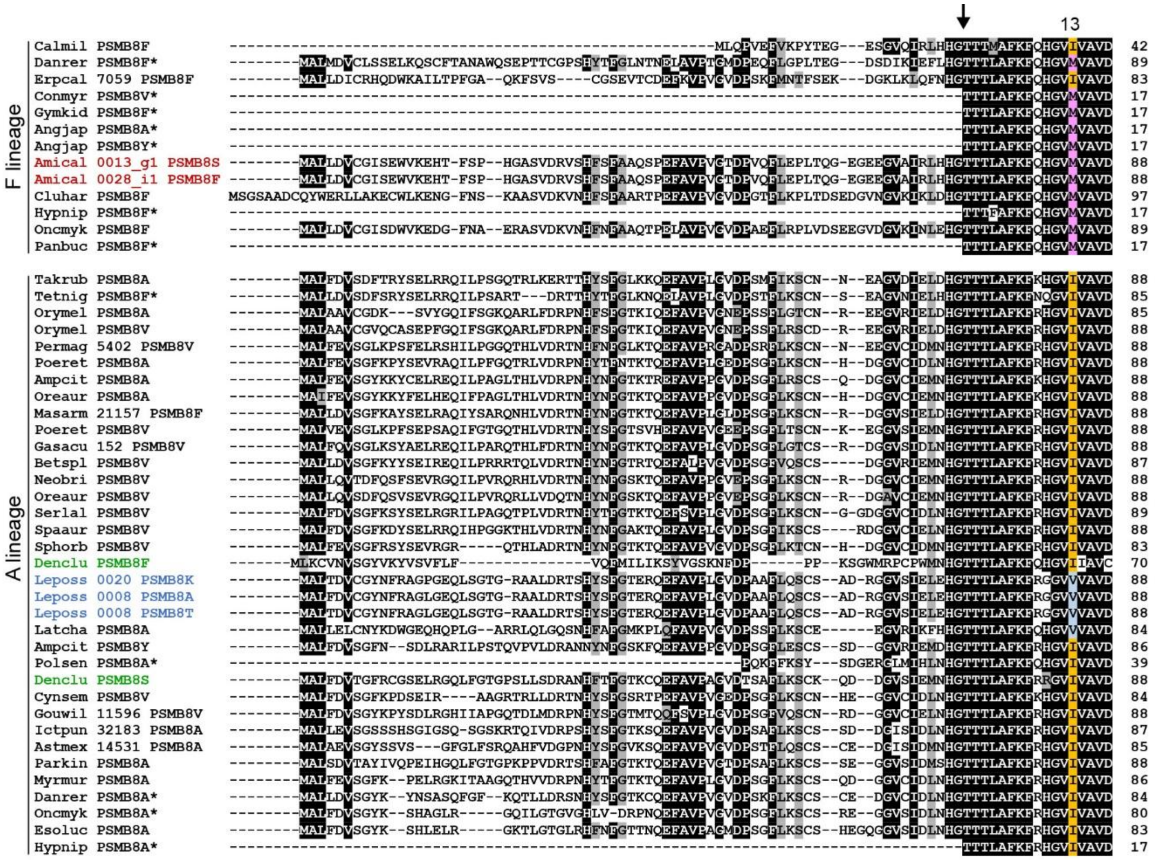
13
F lineage
Calmil PSMB8F
Danrer PSMB8F*
Erpcal 7059 PSMB8F
Conmyr PSMB8V*
Gymkid PSMB8F*
Angjap PSMB8A*
Angjap PSMB8Y*
Amical 0013_g1 PSMB8S
Amical 0028_i1 PSMB8F
Cluhar PSMB8F
Hypnip PSMB8F*
Oncmyk PSMB8F
Panbuc PSMB8F*
A lineage
Takrub PSMB8A
Tetnig PSMB8F*
Orymel PSMB8A
Orymel PSMB8V
Permag 5402 PSMB8V
Poeret PSMB8A
Ampcit PSMB8A
Oreaur PSMB8A
Masarm 21157 PSMB8F
Poeret PSMB8V
Gasacu 152 PSMB8V
Betspl PSMB8V
Neobri PSMB8V
Oreaur PSMB8V
Serlal PSMB8V
Spaaur PSMB8V
Sphorb PSMB8V
Denclu PSMB8F
Leposs 0020 PSMB8K
Leposs 0008 PSMB8A
Leposs 0008 PSMB8T
Latcha PSMB8A
Ampcit PSMB8Y
Polsen PSMB8A*
Denclu PSMB8S
Cynsem PSMB8V
Gouwil 11596 PSMB8V
Ictpun 32183 PSMB8A
Astmex 14531 PSMB8A
Parkin PSMB8A
Myrmur PSMB8A
Danrer PSMB8A*
Oncmyk PSMB8A*
Esoluc PSMB8A
Hypnip PSMB8A*

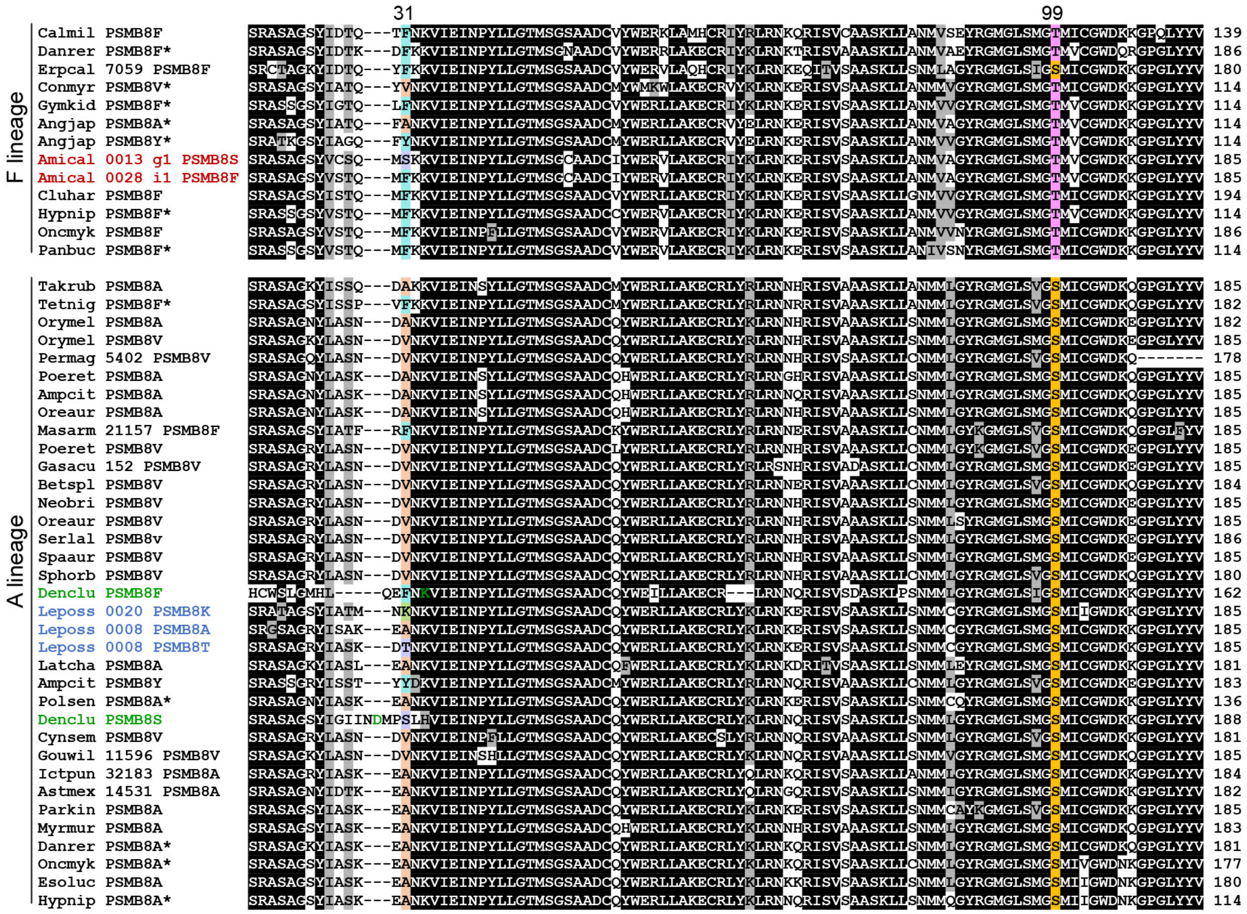

31
99
F lineage
Calmil PSMB8F SRASAGSYIDTQ---TFNKVIEINPYLLGTMSGSAADCVYWERKLAMHCRIYRLRNKQRISVCAASKLLANMVSEYRGMGLSMGTMICGWDKKGPQLYYV 139
Danrer PSMB8F* SRASAGSYIDTK---DFKKVIEINPYLLGTMSGNAADCVYWERRLAKECRIYKLRNKTRISVAAASKLLANMVAEYRGMGLSMGTMVCGWDQRGPGLYYV 186
Erpcal 7059 PSMB8F SRCTAGKYIDTQ---YFKKVIEINPYLLGTMSGSAADCVYWERVLAQHCRIYKLRNKEQITVSAASKLLSNMLAGYRGMGLSIGSMICGWDKKGPGLYYV 180
Conmyr PSMB8V* SRASAGSYIATQ---YVNKVIEINPYLLGTMSGSAADCMYWMKWLAKECRVYKLRNKERISVSAASKLLANMMVGYRGMGLSMGTMICGWDKKGPGLYYV 114
Gymkid PSMB8F* SRASSGSYIGTQ---LFNKVIEINPYLLGTMSGSAADCVYWERLLAKECRIYKLRNKERISVSAASKLLANMVVGYRGMGLSMGTMVCGWDKKGPGLYYV 114
Angjap PSMB8A* SRASAGSYIATQ---FANKVIEINPYLLGTMSGSAADCMYWERLLAKECRVYELRNKERISVSAASKLLANMVAGYRGMGLSMGTMVCGWDKKGPGLYYV 114
Angjap PSMB8Y* SRATKGSYIAGQ---FYNKVIEINPYLLGTMSGSAADCMYWERLLAKECRVYELRNKERISVSAASKLLANMVVGYRGMGLSMGTMVCGWDKKGPGLYYV 114
Amical 0013 g1 PSMB8S SRASAGSYVCSQ---MSKKVIEINPYLLGTMSGCAADCIYWERVLAKECRIYKLRNKERISVSAASKLLANMVAGYRGMGLSMGTMVCGWDKKGPGLYYV 185
Amical 0028 i1 PSMB8F SRASAGSYVSTQ---MFKKVIEINPYLLGTMSGCAADCIYWERVLAKECRIYKLRNKERISVSAASKLLANMVAGYRGMGLSMGTMVCGWDKKGPGLYYV 185
Cluhar PSMB8F SRASAGSYISTQ---MFKKVIEINPYLLGTMSGSAADCVYWERLLAKECRIYKLRNKERISVSAASKLLGNMVVGYRGMGLSMGTMICGWDKKGPGLYYV 194
Hypnip PSMB8F* SRASSGSYVSTQ---MFKKVIEINPYLLGTMSGSAADCCYWERVLAKECRIYKLRNKERISVSAASKLLANMVVGYRGMGLSMGTMVCGWDKKGPGLYYV 114
Oncmyk PSMB8F SRASAGSYVSTQ---MFKKVIEINPFLLGTMSGSAADCVYWERVLAKECRIYKLRNKERISVSAASKLLANMVVNYRGMGLSMGTMICGWDKKGPGLYYV 186
Panbuc PSMB8F* SRASSGSYVSTQ---MFKKVIEINPYLLGTMSGSAADCVYWERVLAKECRIYKLRNKERISVSAASKLLANIVSNYRGMGLSMGTMICGWDKKGPGLYYV 114
A lineage
Takrub PSMB8A SRASAGKYISSQ---DAKKVIEINSYLLGTMSGSAADCMYWERLLAKECRLYRLRNNHRISVAAASKLLANMMLGYRGMGLSVGSMICGWDKQGPGLYYV 185
Tetnig PSMB8F* SRASAGSYISSP---VFKKVIEINPYLLGTMSGSAADCMYWERLLAKECRLYRLRNNRRISVAAASKLLANMMLGYRGMGLSVGSMICGWDKQGPGLYYV 182
Orymel PSMB8A SRASAGNYLASN---DANKVIEINPYLLGTMSGSAADCQYWERLLAKECRLYKLRNNHRISVAAASKLLSNMMLGYRGMGLSMGSMICGWDKEGPGLYYV 182
Orymel PSMB8V SRASAGKYLASN---DVNKVIEINPYLLGTMSGSAADCQYWERLLAKECRLYRLRNNHRISVAAASKLLSNMMLGYRGMGLSMGSMICGWDKEGPGLYYV 185
Permag 5402 PSMB8V SRASAGQYLASN---DVNKVIEINPYLLGTMSGSAADCQYWERLLAKECRLYRLRNNHRISVSAASKLLCNMMLGYRGMGLSVGSMICGWDKQ------- 178
Poeret PSMB8A SRASAGNYLASK---DANKVIEINSYLLGTMSGSAADCQHWERLLAKECRLYRLRNGHRISVAAASKLLSNMMLGYRGMGLSMGSMICGWDKQGPGLYYV 185
Ampcit PSMB8A SRASAGNYLASK---DANKVIEINSYLLGTMSGSAADCQHWERLLAKECRLYRLRNNQRISVAAASKLLSNMMLGYRGMGLSMGSMICGWDKQGPGLYYV 185
Oreaur PSMB8A SRASAGNYLASK---DANKVIEINSYLLGTMSGSAADCQHWERLLAKECRLYRLRNNHRISVAAASKLLSNMMLGYRGMGLSMGSMICGWDKQGPGLYYV 185
Masarm 21157 PSMB8F SRASAGSYIATF---RFNKVIEINPYLLGTMSGSAADCKYWERLLAKECRLYRLRNNERISVAAASKLLCNMMLGYKGMGLSVGSMICGWDKQGPGLFYV 185
Poeret PSMB8V SRASAGRYLASN---DVNKVIEINPYLLGTMSGSAADCLYWERLLAKECRLYRLRNNHRISVAAASKLLCNMMLGYKGMGLSVGSMICGWDKEGPGLYYV 185
Gasacu 152 PSMB8V SRASAGRYLASN---DVNKVIEINPYLLGTMSGSAADCQYWERLLAKECRLYRLRSNHRISVADASKLLCNMMLGYRGMGLSMGSMICGWDKEGPGLYYV 185
Betspl PSMB8V SRASAGRYLASN---DVNKVIEINPYLLGTMSGSAADCQYWERLLAKECRLYRLRNNERISVAAASKLLCNMMLGYRGMGLSVGSMICGWDKQGPGLYYV 184
Neobri PSMB8V SRASAGRYLASN---DVNKVIEINPYLLGTMSGSAADCQYWERLLAKECRLYRLRNNHRISVAAASKLLSNMMLGYRGMGLSMGSMICGWDKEGPGLYYV 185
Oreaur PSMB8V SRASAGRYLASN---DVNKVIEINPYLLGTMSGSAADCQYWERLLAKECRLYRLRNNHRISVAAASKLLSNMMLSYRGMGLSMGSMICGWDKEGPGLYYV 185
Serlal PSMB8v SRASAGRYLASN---DVNKVIEINPYLLGTMSGSAADCQYWERLLAKECRLYRLRNNHRISVAAASKLLSNMMLGYRGMGLSMGSMICGWDKEGPGLYYV 186
Spaaur PSMB8V SRASAGRYLASN---DVNKVIEINPYLLGTMSGSAADCQYWERLLAKECRLYRLRNNHRISVAAASKLLSNMMLGYRGMGLSMGSMICGWDKEGPGLYYV 185
Sphorb PSMB8V SRASAGRYLASN---DVNKVIEINPYLLGTMSGSAADCQYWERLLAKECRLYRLRNNHRISVAAASKLLCNMMLGYRGMGLSVGSMICGWDKQGPGLYYV 180
Denclu PSMB8F HCWSLGMHL-----QEFNKVIEINPYLLGTMSGSAADCQYWEILLAKECR---LRNNQRISVSDASKLPSNMMLGYRGMGLSIGSMICGWDKKGPGLYYV 162
Leposs 0020 PSMB8K SRATAGSYIATM---NKNKVIEINPYLLGTMSGSAADCQYWERLLAKECRLYKLRNKERISVSAASKLLSNMMCGYRGMGLSMGSMIIGWDKKGPGLYYV 185
Leposs 0008 PSMB8A SRGSAGRYISAK---EANKVIEINPYLLGTMSGSAADCQYWERLLAKECRLYKLRNKERISVSAASKLLSNMMCGYRGMGLSMGSMICGWDKKGPGLYYV 185
Leposs 0008 PSMB8T SRASAGRYIASK---DTNKVIEINPYLLGTMSGSAADCQYWERLLAKECRLYKLRNKERISVSAASKLLSNMMCGYRGMGLSMGSMICGWDKKGPGLYYV 185
Latcha PSMB8A SRASAGKYIASL---EANKVIEINPYLLGTMSGSAADCQFWERLLAKECRLYKLRNKDRITVSAASKLLSNMMLEYRGMGLSMGSMICGWDKKGPGLYYV 181
Ampcit PSMB8Y SRASSGRYISST---YYDKVIEINPYLLGTMSGSAADCMYWERLLAKECRLYRLRNNQRISVAAASKLLCNMMLGYRGMGLSVGSMICGWDKEGPGLYYV 183
Polsen PSMB8A* SRASAGNYIASK---EANKVIEINPYLLGTMSGSAADCQYWERLLAKECRLYKLRNKERISVSAASKLLSNMMCQYRGMGLSMGSMICGWDKKGPGLYYV 136
Denclu PSMB8S SRASAGSYIGIINDMPSLHVIEINPYLLGTMSGSAADCQYWERLLAKECRLYKLRNNQRISVSAASKLLSNMMLGYRGMGLSMGSMICGWDKKGPGLYYV 188
Cynsem PSMB8V SRASAGRYLASN---DVNKVIEINPFLLGTMSGSAADCQYWERLLAKECSLYRLRNNQRISVAAASKLLSNMMLGYRGMGLSVGSMICGWDKKGPGLYYV 181
Gouwil 11596 PSMB8V SRASAGKYLASN---DVNKVIEINSHLLGTMSGSAADCQYWERLLAKECRLYKLRNNQRISVAAASKLLSNMMVGYRGMGLSMGSMICGWDKQGPGLYYV 185
Ictpun 32183 PSMB8A SRASAGRYIASK---EANKVIEINPYLLGTMSGSAADCQYWERLLAKECRLYQLRNKQRISVSAASKLLANMMLGYRGMGLSMGSMICGWDKKGPGLYYV 184
Astmex 14531 PSMB8A SRASAGNYIDTK---EANKVIEINPYLLGTMSGSAADCQYWERLLAKECRLYQLRNGQRISVSAASKLLSNMMIGYRGMGLSMGSMICGWDKKGPGLYYV 182
Parkin PSMB8A SRASAGSYIASK---EANKVIEINPYLLGTMSGSAADCQYWERLLAKECRLYKLRNKERISVSAASKLLSNMMCAYKGMGLSVGSMICGWDKKGPGLYYV 185
Myrmur PSMB8A SRASAGSYIASK---EANKVIEINPYLLGTMSGSAADCQHWERLLAKECRLYKLRNNHRISVAAASKLLSNMMLGYRGMGLSMGSMICGWDKQGPGLYYV 183
Danrer PSMB8A* SRASAGKYIASK---EANKVIEINPYLLGTMSGSAADCQYWERLLAKECRLYKLRNKQRISVSAASKLLSNMMLGYRGMGLSMGSMICGWDKQGPGLYYV 181
Oncmyk PSMB8A* SRASAGSYIASK---EANKVIEINPYLLGTMSGSAADCQYWERLLAKECRLYKLRNKQRISVSAASKLLCNMMLGYRGMGLSMGSMIVGWDNKGPGLYYV 177
Esoluc PSMB8A SRASAGSYIASK---EANKVIEINPYLLGTMSGSAADCQYWERLLAKECRLYKLRNKKRISVSAASKLLSNMMLGYRGMGLSMGSMIIGWDNKGPGLYYV 180
Hypnip PSMB8A* SRASAGSYIASK---EANKVIEINPYLLGTMSGSAADCQYWERLLAKECRLYKLRNNQRISVSAASKLLSNMMQGYRGMGLSMGSMIIGWDNKGPGLYYV 114

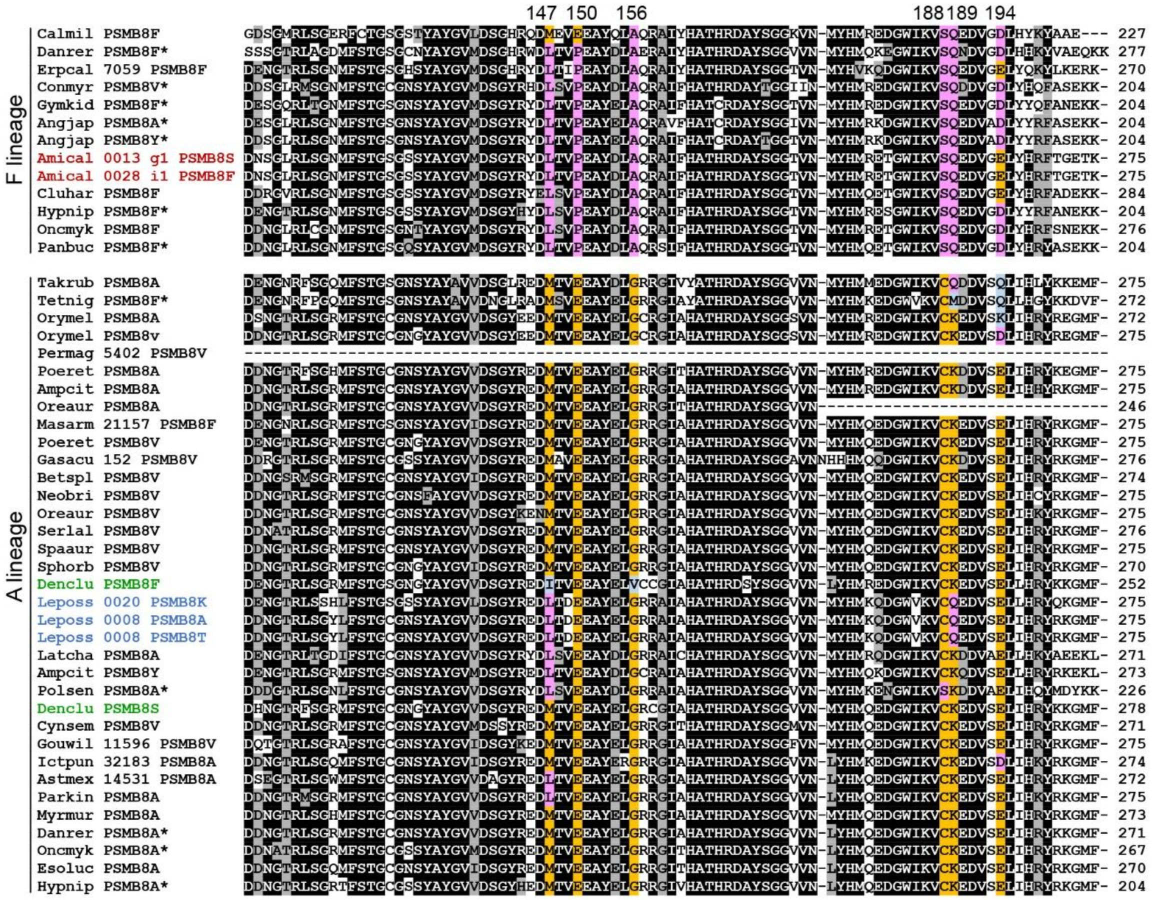

Supplementary Figure S3. Alignment of select PSMB8 sequences.

A lineage and F lineage PSMB8 sequences from major Actinopterygian lineages were aligned using Clustal Omega (Sievers and Higgins 2021). Efforts were made to include major radiations as well as examples of unusual sequence types (residue 31). Positions that are ≥70% identical are shaded black and those that are structurally related are shaded gray. The predicted cleavage site which produces the mature protein is indicated with an arrow. The eight residues described as predictive for the A and F lineages - 13, 99, 147, 150, 156, 188, 189 and 194 (Noro and Nonaka 2014) - are shaded gold and pink, respectively (summarized in **Table 1**). Residues that do not match either lineage are shaded blue. Residue 31, which defines the PSMB8 type is shaded orange (A type with A or V), aqua (F type with F or Y), purple (S type with S or T) or green (K type). Sequence identifiers for ruddy bowfin and longnose gar are indicated with red and blue text— this is to highlight the novel PSMB8 S and K types observed in these holostean lineages. Sequence identifiers for two PSMB8 types from the divergent denticle herring (Denclu) are indicated with green text — note that, as both forms appear to have insertions or deletions between position 1 and 31 of the mature protein, position 31 of these sequences do not align with position 31 of all other species in this analysis making the assignment of a PSMB8 type challenging. Our nomenclature used denticle herring residues that align with position 31 of other species (F and S). In contrast, the actual residues in position 31 are K and D (green text). Sequences previously reported by Noro and Nonaka (2014) are indicated by asterisks (*). All sequences and display identifiers are provided in **Supplementary Tables S1 - S4**.

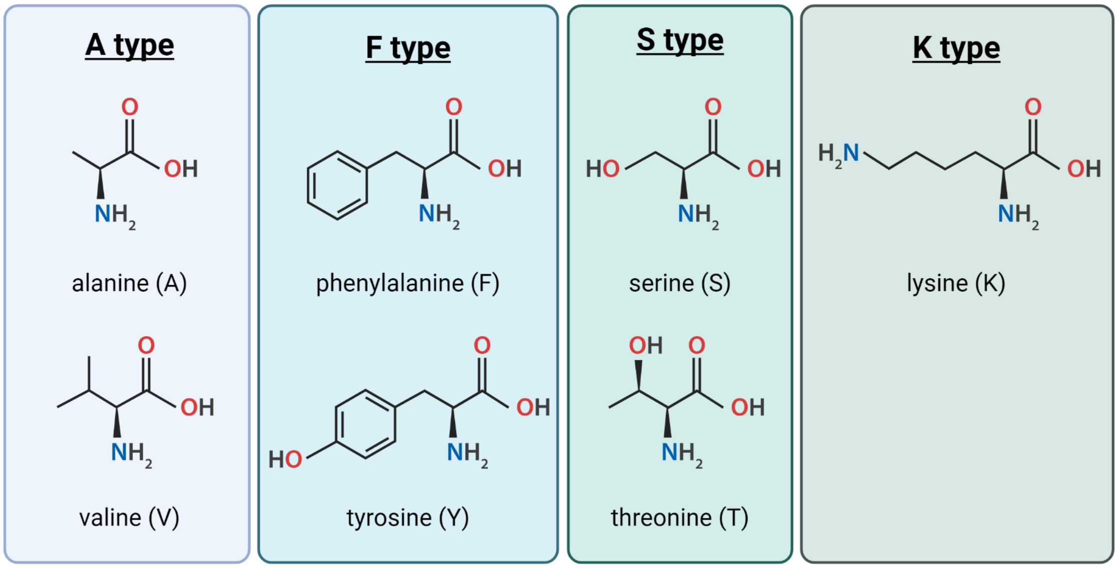

Supplementary Figure S4. Thirty-first residues of PSMB8 types.

Amino acids present in the 31st position of the mature PSMB8 protein define the “type”. Figure created in BioRender. Yoder, J. (2025) https://BioRender.com/w43r864.

```
                         E263-L299                              P375 - M420
Amical_0013_TAP1a_v1     LVFSSVLKQEIAFFDSMHTGDIVSRITTDTNTMSESL  KTVRSFANEEGEARRYAERLEETYTHNKTEAAAYALSTWTNSLSSL
Amical_0013_TAP1a_v2     LVFSSVLKQEIAFFDSMHTGDIVSRITTDTNTMSESL  KTVRSFANEEGEARRYAERLEETYTHNKTEAAAYALSTWTNSLSSL
Amical_0016_TAP1a_v1     LVFSSVLKQEIAFFDSMHTGDIVSRITTDTNTMSESL  KTVRSFANEEGEARRYAERLEETYTHNKTEAAAYALSTWTNSLSSL
Amical_0016_TAP1a_v2     LVFSSVLKQEIAFFDSMHTGDIVSRITTDTNTMSESL  KTVRSFANEEGEARRYAERLEETYTHNKTEAAAYALSTWTNSLSSL
Amical_0017_TAP1a_v2     LVFSSVLKQEIAFFDSMHTGDIVSRITTDTNTMSESL  KTVRSFANEEGEARRYAERLEETYTHNKTEAAAYALSTWTNSLSSL
Amical_0018_TAP1a_v2     LVFSSVLKQEIAFFDSMHTGDIVSRITTDTNTMSESL  KTVRSFANEEGEARRYAERLEETYTHNKTEAAAYALSTWTNSLSSL
Amical_0026_TAP1a_v1     LVFSSVLKQEIAFFDSMHTGDIVSRITTDTNTMSESL  KTVRSFANEEGEARRYAERLEETYTHNKTEAAAYALSTWTNSLSSL
Amical_0027_TAP1a_v2     LVFSSVLKQEIAFFDSMHTGDIVSRITTDTNTMSESL  KTVRSFANEEGEARRYAERLEETYTHNKTEAAAYALSTWTNSLSSL
Amical_0028_TAP1a_v2     LVFSSVLKQEIAFFDSMHTGDIVSRITTDTNTMSESL  KTVRSFANEEGEARRYAERLEETYTHNKTEAAAYALSTWTNSLSSL
Amioce_0035_TAP1a_v1     LVFSSVLKQEIAFFDSMHTGDIVSRITTDTNTMSESL  KTVRSFANEEGEARRYAERLEETYTHNKTEAAAYALSTWTNSLSSL
Amioce_0036_TAP1a_v2     LVFSSVLKQEIAFFDSMHTGDIVSRITTDTNTMSESL  KTVRSFANEEGEARRYAERLEETYTHNKTEAAAYALSTWTNSLSSL
Amioce_0037_TAP1a_v1     LVFSSVLKQEIAFFDSMHTGDIVSRITTDTNTMSESL  KTVRSFANEEGEARRYAERLEETYTHNKTEAAAYALSTWTNSLSSL
Amioce_0038_TAP1a_v1     LVFSSVLKQEIAFFDSMHTGDIVSRITTDTNTMSESL  KTVRSFANEEGEARRYAERLEETYTHNKTEAAAYALSTWTNSLSSL
Amioce_0039_TAP1a_v1     LVFSSVLKQEIAFFDSMHTGDIVSRITTDTNTMSESL  KTVRSFANEEGEARRYAERLEETYTHNKTEAAAYALSTWTNSLSSL
Amioce_0040_TAP1a_v2     LVFSSVLKQEIAFFDSMHTGDIVSRITTDTNTMSESL  KTVRSFANEEGEARRYAERLEETYTHNKTEAAAYALSTWTNSLSSL

Amical_0013_TAP1b_v2     LVFSSVLKQEIAFFDSMHTGDIVSRITTDTNTMSESL  KTVRSFANEEGEARRYAERLEETYTHNKTEAAAYALSTWTNSLSSL
Amical_0015_TAP1b_v1     LVFSSVLKQEIAFFDSMHTGDIVSRITTDTNTMSESL  KTVRSFANEEGEARRYAERLEETYTHNKTEAAAYALSTWTNSLSSL
Amical_0018_TAP1b_v2     LVFSSVLKQEIAFFDSMHTGDIVSRITTDTNTMSESL  KTVRSFANEEGEARRYAERLEETYTHNKTEAAAYALSTWTNSLSSL
Amioce_0035_TAP1b_v1     LVFSSVLKQEIAFFDSMHTGDIVSRITTDTNTMSESL  KTVRSFANEEGEARRYAERLEETYTHNKTEAAAYALSTWTNSLSSL
Amioce_0038_TAP1b_v1     LVFSSVLKQEIAFFDSMHTGDIVSRITTDTNTMSESL  KTVRSFANEEGEARRYAERLEETYTHNKTEAAAYALSTWTNSLSSL
Amioce_0039_TAP1b_v1     LVFSSVLKQEIAFFDSMHTGDIVSRITTDTNTMSESL  KTVRSFANEEGEARRYAERLEETYTHNKTEAAAYALSTWTNSLSSL

Zebrafish_Tap1           LVFQAVLKQDIAFFDKATTGDIVSRITTDTNTMSESL  KTVRSFANEDGETERYRKCLEDTFALNKVEAAAYAVSTWTNSMSSL
Human_TAP1               EVFGAVLRQETEFFQQNQTGNIMSRVTEDTSTLSDSL  PTVRSFANEEGEAQKFREKLQEIKTLNQKEAVAYAVNSWTTSISGM


                         Q453 - R487
Amical_0013_TAP1a_v1     QFTSAVEVLLNYYPHVKKAIGASEKIFELMDREPL
Amical_0013_TAP1a_v2     QFTSAVEVLLNYYPHVKKAIGASEKIFELMDRELL
Amical_0016_TAP1a_v1     QFTSAVEVLLNYYPHVKKAIGASEKIFELMDREPL
Amical_0016_TAP1a_v2     QFTSAVEVLLNYYPHVKKAIGASEKIFELMDRELL
Amical_0017_TAP1a_v2     QFTSAVEVLLNYYPHVKKAIGASEKIFELMDRELL
Amical_0018_TAP1a_v2     QFTSAVEVLLNYYPHVKKAIGASEKIFELMDRELL
Amical_0026_TAP1a_v1     QFTSAVEVLLNYYPHVKKAIGASEKIFELMDREPL
Amical_0027_TAP1a_v2     QFTSAVEVLLNYYPHVKKAIGASEKIFELMDRELL
Amical_0028_TAP1a_v2     QFTSAVEVLLNYYPHVKKAIGASEKIFELMDRELL
Amioce_0035_TAP1a_v1     QFTSAVEVLLNYYPHVKKAIGASEKIFELMDREPL
Amioce_0036_TAP1a_v2     QFTSAVEVLLNYYPHVKKAIGASEKIFELMDRELL
Amioce_0037_TAP1a_v1     QFTSAVEVLLNYYPHVKKAIGASEKIFELMDREPL
Amioce_0038_TAP1a_v1     QFTSAVEVLLNYYPHVKKAIGASEKIFELMDREPL
Amioce_0039_TAP1a_v1     QFTSAVEVLLNYYPHVKKAIGASEKIFELMDREPL
Amioce_0040_TAP1a_v2     QFTSAVEVLLNYYPHVKKAIGASEKIFELMDRELL

Amical_0013_TAP1b_v2     QFTSAVEVLLNYYPHVKKAIGASEKIFELMDRELL
Amical_0015_TAP1b_v1     QFTSAVEVLLNYYPHVKKAIGASEKIFELMDREPL
Amical_0018_TAP1b_v2     QFTSAVEVLLNYYPHVKKAIGASEKIFELMDRELL
Amioce_0035_TAP1b_v1     QFTSAVEVLLNYYPHVKKAIGASEKIFELMDREPL
Amioce_0038_TAP1b_v1     QFTSAVEVLLNYYPHVKKAIGASEKIFELMDREPL
Amioce_0039_TAP1b_v1     QFTSAVEVLLNYYPHVKKAIGASEKIFELMDREPL

Zebrafish_Tap1           QFTSAVEVLMSYWPHVKKAVGASEKIFEYVDRKPD
Human_TAP1               QFTQAVEVLLSIYPRVQKAVGSSEKIFEYLDRTPR
```

Supplementary Figure S5. Essential TAP1 residues from individual bowfin.

Bowfin TAP1 regions encoding residues related to functionality were aligned using Clustal Omega (Sievers and Higgins 2021). Human (GenBank NP_000584.3) and zebrafish (GenBank XP_002665053.1) TAP1 sequences are included for reference with amino acid numbering from human TAP1 shown above. Positions that are ≥90% identical are shaded black. Positions related to functionality (263), peptide sensing (282, 284, 287, 288), and substrate specificity (296, 408, 459) are shaded in blue, yellow, and green respectively (Lehnert and Tampé 2017).

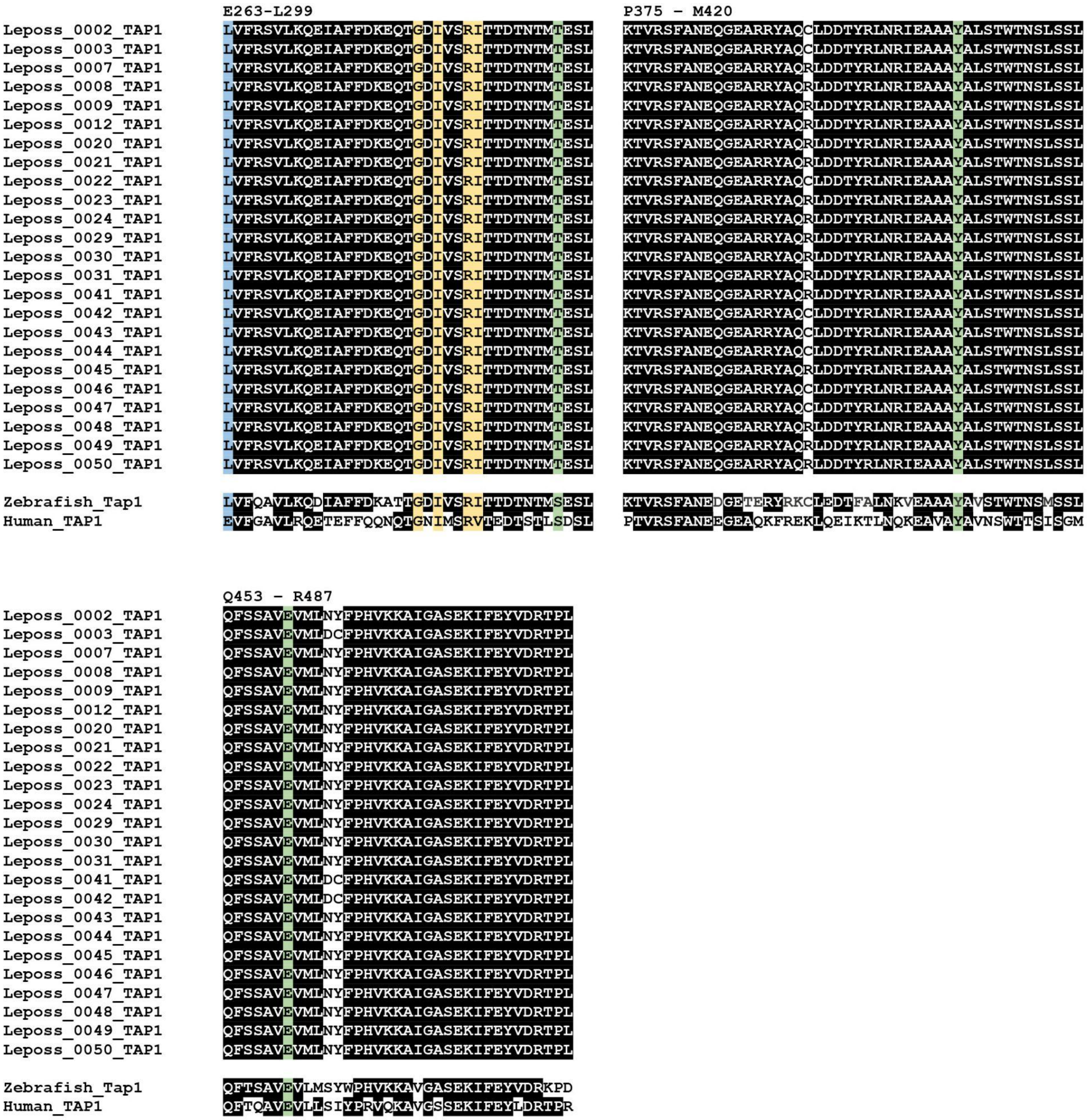

Supplementary Figure S6. Essential TAP1 residues from individual longnose gar.

Longnose gar TAP1 regions encoding residues related to functionality were aligned using Clustal Omega (Sievers and Higgins 2021). Human (GenBank NP_000584.3) and zebrafish (GenBank XP_002665053.1) TAP1 sequences are included for reference with amino acid numbering from human TAP1 shown above. Positions that are ≥90% identical are shaded black. Positions related to functionality (263), peptide sensing (282, 284, 287, 288), and substrate specificity (296, 408, 459) are shaded in blue, yellow, and green respectively (Lehnert and Tampé 2017).

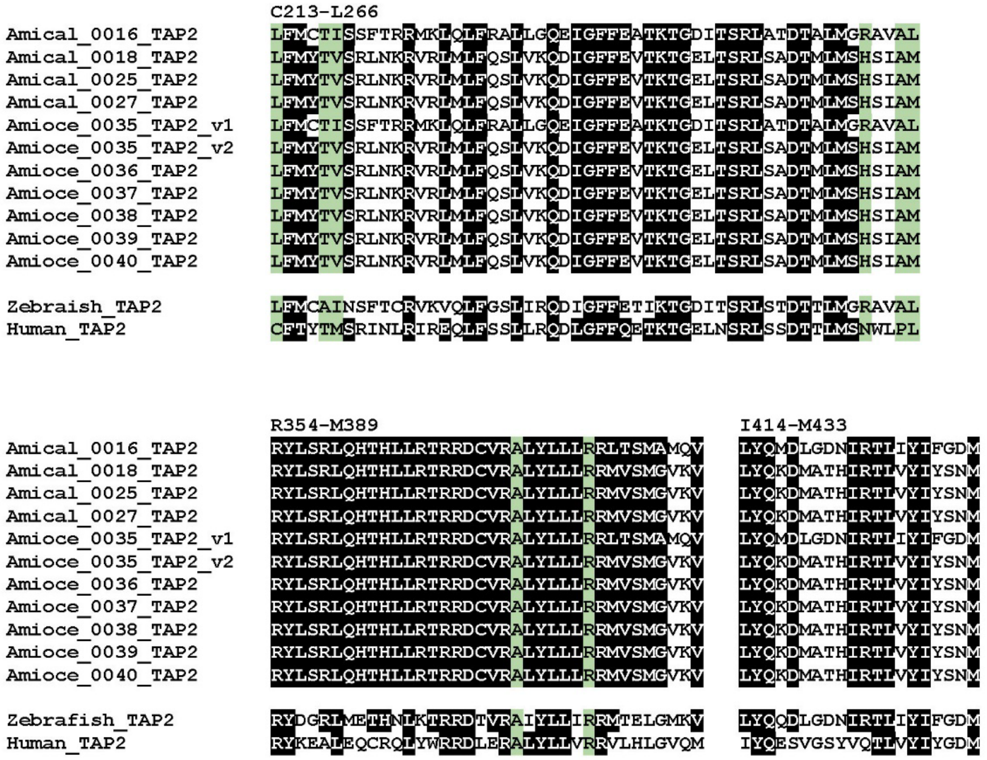

Supplementary Figure S7. Essential TAP2 residues from individual bowfin.

Bowfin TAP2 regions encoding residues related to functionality were aligned using Clustal Omega (Sievers and Higgins 2021). Human (GenBank NP_000535.3) and zebrafish (GenBank NP_001006594.1) TAP2 sequences are included for reference with amino acid numbering from human TAP2 shown above. Positions that are ≥90% identical are shaded black. Positions related to substrate specificity (213, 217, 218, 262, 265, 266, 374, 380) are shaded green and regions associated with peptide binding are included (354-389 and 414-433) (Lehnert and Tampé 2017).

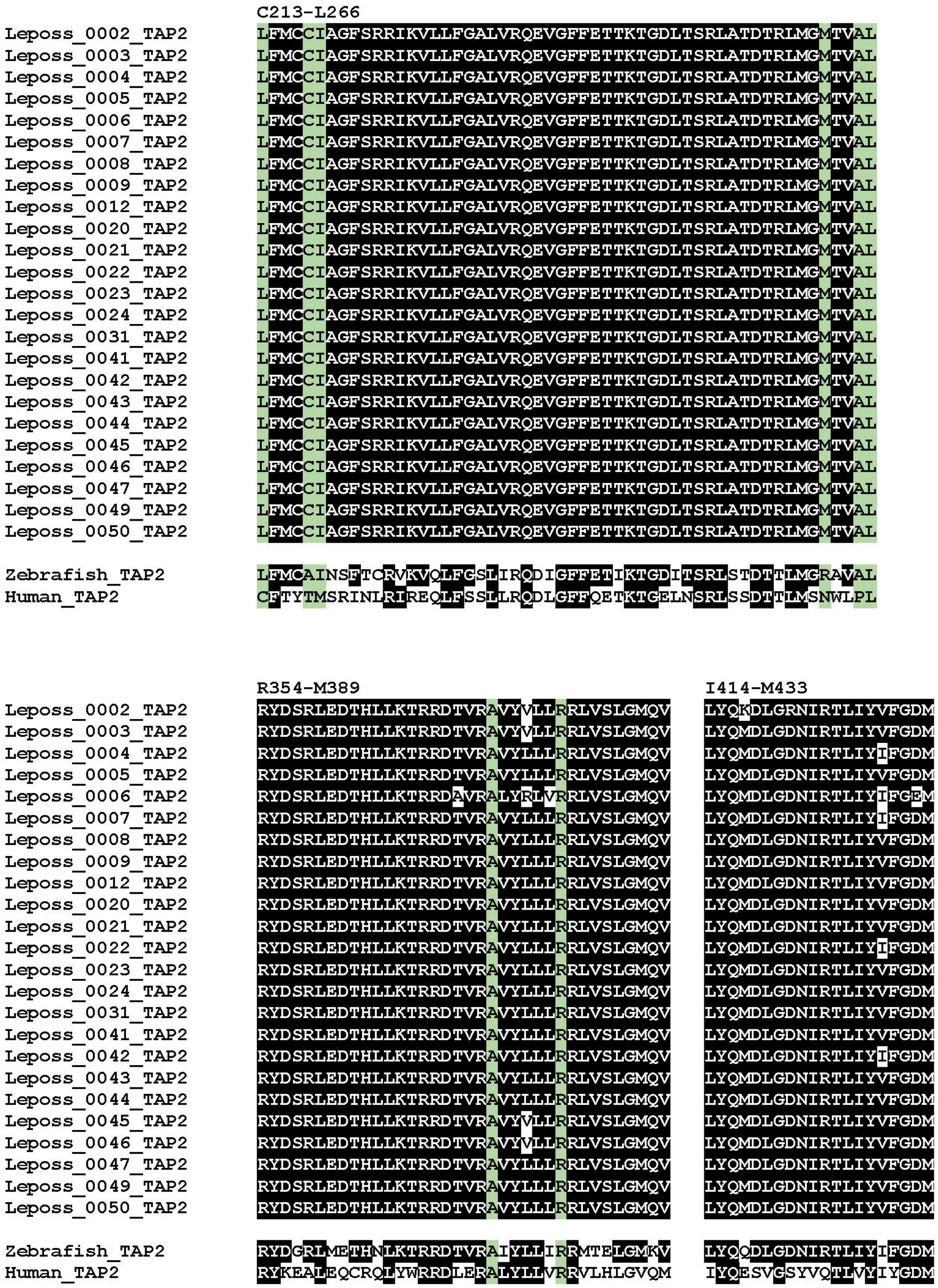

Supplementary Figure S8. Essential TAP2 residues from individual longnose gar.

Longnose gar TAP2 regions encoding residues related to functionality were aligned using Clustal Omega (Sievers and Higgins 2021). Human (GenBank NP_000535.3) and zebrafish (GenBank NP_001006594.1) TAP2 sequences are included for reference with amino acid numbering from human TAP2 shown above. Positions that are ≥90% identical are shaded black. Positions related to substrate specificity (213, 217, 218, 262, 265, 266, 374, 380) are shaded green and regions associated with peptide binding are included (354-389 and 414-433) (Lehnert and Tampé 2017).

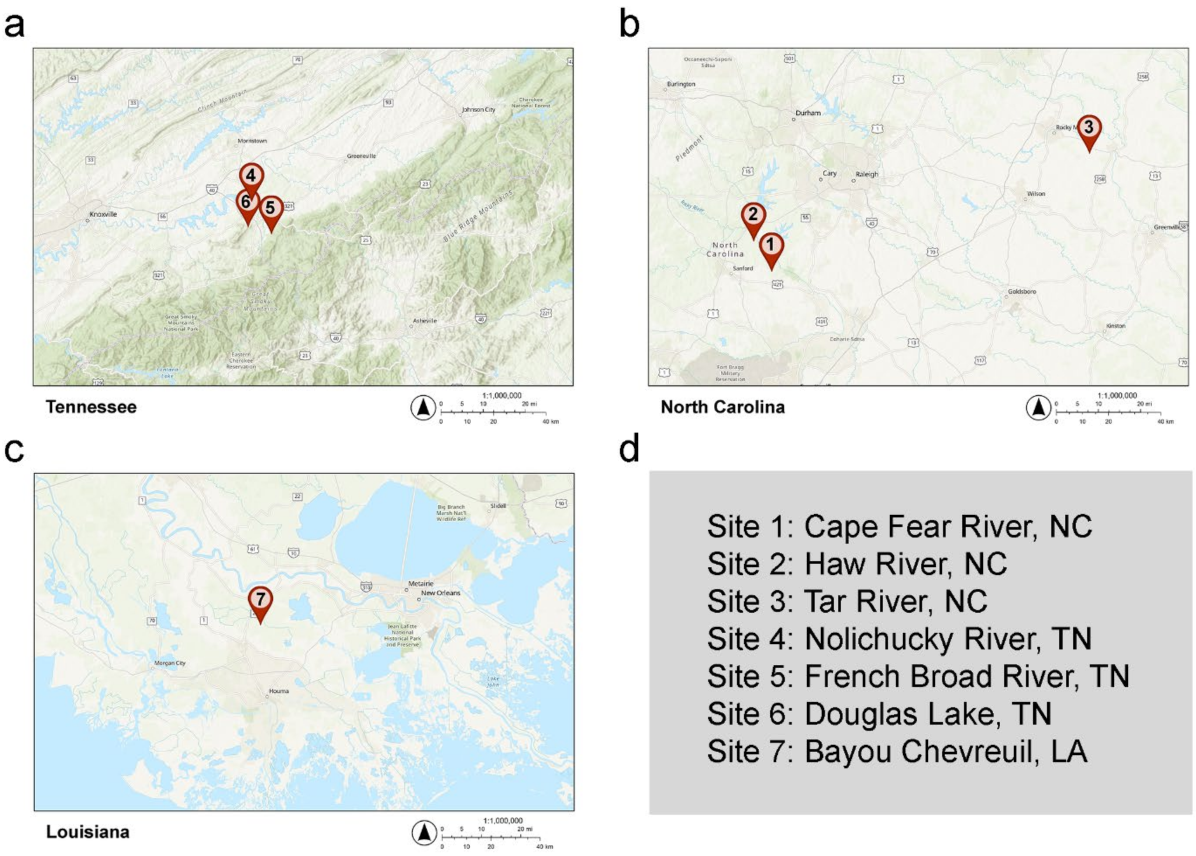

Supplementary Figure S9. Collection sites for bowfin and longnose gar.

The locations where bowfin and longnose gar were collected are indicated in maps of **a** Tennessee, **b** North Carolina, and **c** Louisiana. Locations are listed in **d** with coordinates provided in **Supplementary Tables S9 and S10**. Maps were generated using ArcGIS Online software.

References.

Bi X, Wang K, Yang L, Pan H, Jiang H, Wei Q, Fang M, Yu H, Zhu C, Cai Y, et al. 2021. Tracing the genetic footprints of vertebrate landing in non-teleost ray-finned fishes. *Cell* 184:1377–1391.e14.

Braasch I, Gehrke AR, Smith JJ, Kawasaki K, Manousaki T, Pasquier J, Amores A, Desvignes T, Batzel P, Catchen J, et al. 2016. The spotted gar genome illuminates vertebrate evolution and facilitates human-teleost comparisons. *Nat. Genet.* 48:427–437.

Lehnert E, Tampé R. 2017. Structure and dynamics of antigenic peptides in complex with TAP. *Front. Immunol.* 8:10.

Noro M, Nonaka M. 2014. Evolution of dimorphisms of the proteasome subunit beta type 8 gene (PSMB8) in basal ray-finned fish. *Immunogenetics* 66:325–334.

Sievers F, Higgins DG. 2021. The Clustal Omega Multiple Alignment Package. *Methods Mol. Biol.* 2231:3–16.
